# Epidemiology of sporadic and outbreak-associated hepatitis A infections in Ontario, Canada: A descriptive summary, 2015–2022

**DOI:** 10.14745/ccdr.v50i09a05

**Published:** 2024-09-05

**Authors:** Katherine Paphitis, Janica A Adams, Christine Navarro

**Affiliations:** 1 Health Protection, Public Health Ontario, Toronto, ON

**Keywords:** disease outbreaks, epidemiology, hepatitis A, immunization, public health surveillance

## Abstract

**Background:**

Hepatitis A is a disease of public health significance that typically causes acute, self-limiting infection. Understanding the risk factors and demographics associated with individual infections and outbreaks can guide public health communication and interventions.

**Objective:**

To assess the number of hepatitis A cases and outbreaks in Ontario from January 1, 2015, to November 22, 2022, and to identify common risk factors associated with sporadic and outbreak-associated infections in Ontario.

**Methods:**

Confirmed and probable hepatitis A cases reported between January 1, 2015, and November 22, 2022, were extracted from the Ontario electronic reporting system. Descriptive analyses were used to summarize and compare risk factors reported by sporadic and outbreak-associated hepatitis A cases. Annual rates of infection for individual public health units were calculated using annual population estimates for Ontario health regions.

**Results:**

During the study period, 938 cases of hepatitis A were reported in Ontario (an average annual rate of 0.9 cases per 100,000 population), with 31.3% (n=294) of cases linked to one of 18 unique outbreaks of hepatitis A. Four of 13 local outbreaks were associated with elementary school settings. Reported risk factors differed between sporadic cases (predominantly travel-related) and cases linked to known outbreaks (anal-oral contact, illicit drug use, diapering/assisting in toileting, close contact with a case). Rates of sporadic infection differed across public health units in Ontario over the study period.

**Conclusion:**

Public health interventions that aim to increase awareness of hepatitis A risk factors and increase vaccine uptake among those at increased risk of exposure could help to reduce the incidence of both locally acquired and travel-related sporadic infections and outbreaks.

## Introduction

Hepatitis A is typically spread via the fecal-oral route and through direct or indirect contact (including anal-oral contact) or ingestion of contaminated food, typically causing acute, self-limiting infection in those who are infected (1). Following exposure to hepatitis A virus (HAV), signs and symptoms typically develop within 28–30 days, although symptoms may occur 15–50 days following exposure (1). Transmission of infection can occur from two weeks prior to symptom onset and up to seven days after onset of jaundice; thus, transmission of infection may occur before a case is aware they are ill (1). While children under six years of age are usually asymptomatic, severity of infection increases with age (1–4).

Hepatitis A incidence is low in developed countries, such as Canada, where most individuals have access to clean water and adequate sanitation (3). Individuals considered to be at increased risk of exposure to hepatitis A in developed countries include men who have sex with men (MSM), people who use drugs (including people who inject drugs), household or sexual contacts of a confirmed case, individuals experiencing homelessness, individuals anticipating close contact with international adoptees and travellers to HAV endemic areas (3,5–7). Hepatitis A virus exposure may also occur through the ingestion of contaminated food, including ready-to-eat foods, shellfish and foods imported from areas with high hepatitis A endemicity (1,3,8–11).

In Ontario, all confirmed and probable cases of hepatitis A are reported to local public health units (PHUs) for investigation (12). Through case interviews using a standardized questionnaire, PHUs collect information on symptoms, medical and behavioural risk factors and relevant exposures during the incubation period (13). Confirmed cases are those with laboratory confirmation of infection (a serum or plasma sample positive for HAV IgM antibody) without recent vaccination for hepatitis A and either acute symptomatic illness or an epidemiologic link to a confirmed case (1). Probable cases are those with acute illness and an epidemiologic link to a confirmed case, but without laboratory confirmation of infection (1). Where two or more cases share a common exposure, an outbreak may be declared. Multijurisdictional outbreaks involve more than one PHU and in some situations, for example, outbreaks linked to consumption of a widely distributed food product, a national outbreak may be declared.

Case management of confirmed and probable cases includes providing education on disease transmission and prevention, excluding cases who work in high-risk settings (such as food handlers, childcare staff and healthcare workers) from work until 14 days after symptom onset or seven days after the onset of jaundice and recommending post-exposure prophylaxis for household and close contacts of HAV cases to minimize risk of transmission (1).

In Ontario, HAV vaccination is not part of the routine childhood immunization schedule but is available to travellers (for a fee) and is publicly funded for individuals at high risk of exposure or severe outcomes, including MSM, people who inject drugs and individuals with chronic liver disease, including hepatitis B and C (14). While individuals at high risk of exposure to hepatitis A are eligible to receive two doses of publicly funded vaccine as a means of primary prevention, a single dose of vaccine may be offered to contacts of cases as post-exposure prophylaxis (1,14,15).

This study aimed to assess the number of reported hepatitis A cases and outbreaks in Ontario from January 1, 2015, to November 22, 2022, and to identify and to compare demographics and reported risk factors for sporadic and outbreak-associated cases. Awareness of specific risk factors associated with sporadic infections and outbreaks can help to target public health communication and interventions aimed at disease prevention.

## Methods

Cases meeting the confirmed or probable case definition for hepatitis A infection in Ontario and reported via the integrated Public Health Information System (iPHIS) by local PHUs from January 1, 2015, to November 22, 2022, were extracted for analyses. Data for 2022 were incomplete as data extraction was performed on November 22, 2022, in response to an internal data request and reanalyzed as a convenience sample. Where case onset date was unavailable, the episode date was used as a proxy per the following hierarchy: onset date, followed by specimen collection date, then laboratory test date, then reported date. Cases were categorized by the reporting PHU as outbreak-confirmed if a common exposure or contact with an infectious case of hepatitis A was known to have occurred, or as sporadic if no linkages to other cases or common exposures were identified at the time of initial case investigation. Outbreak-associated case counts were compared to published outbreak summaries for known outbreaks and, where observed to be misclassified as sporadic in the surveillance system, cases were reassigned to the correct outbreak.

Descriptive analyses were performed using SAS Enterprise Guide 8.2 (SAS Institute Inc., Cary, North Carolina) and Microsoft Excel 2013 (Redmond, Washington). Case data included age, gender (reported as male, female, transgender or unknown), risk factor/exposure information and PHU (based on home address). Annual rates of hepatitis A per 100,000 population were calculated for each PHU using annual population estimates for health regions in Ontario (16). Infection rates were calculated for each PHU (by year and averaged over the study period) to determine which PHUs had the highest rates of sporadic hepatitis A infections. Responses for individual risk factors were assessed separately for sporadic and outbreak-associated cases to explore associations between age or gender and individual risk factors. The Wilcoxon-Mann-Whitney test, Fisher’s exact test or Mantel-Haenszel chi-square test were used to assess the significance of differences between outbreak-confirmed and sporadic hepatitis A cases and case demographics or risk factors (as applicable). Statistical significance for all analyses was 5% (α=0.05).

Research ethics committee approval was not required for this project as the activities described here are considered routine surveillance at Public Health Ontario.

## Results

### Hepatitis A cases and outbreaks

**Figure 1:**
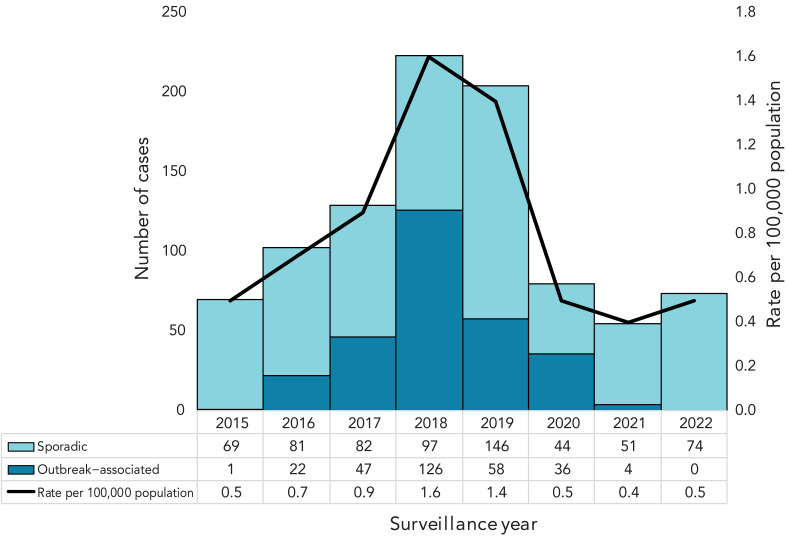
Number of sporadic and outbreak-associated cases of hepatitis A and rates of infection (per 100,000 population) in Ontario, Canada (n=938), January 1, 2015, to November 22, 2022

A total of 938 hepatitis A cases (n=917 confirmed, n=21 probable) were reported from all 34 PHUs in Ontario between January 1, 2015, and November 22, 2022, representing an average annual rate of 0.9 cases per 100,000 population. Of the reported cases, 39 cases that were entered into iPHIS as sporadic but known to be linked to an outbreak of hepatitis A were reassigned to the correct outbreak number. Subsequent analyses were based on corrected sporadic and outbreak-associated case counts. Most cases (68.7%, n=644) were reported as sporadic by the investigating PHU and 31.3% (n=294) were linked to an outbreak of hepatitis A. The number of sporadic and outbreak-associated cases generally increased each year prior to the COVID-19 pandemic (**Figure 1**). The number of reported outbreaks also generally increased each year, with one outbreak in each of 2015 and 2016, three in 2017, two in 2018 and five in each of 2019 and 2020 before declining during pandemic-impacted years.

There was a significant association between sporadic versus outbreak-associated cases and age group (*p*<0.0001). Almost two thirds of sporadic cases were younger than 30 years of age (n=421, 65.4%) compared to 36.7% (n=108) of outbreak-associated cases (**Table 1**).

**Table 1:** Age and gender distribution for confirmed and probable cases of hepatitis A reported in Ontario, Canada, January 1, 2015, to November 22, 2022

Characteristics	Outbreak-associated cases (number, %)	Sporadic cases (number, %)	Total cases (number, %)	*p*-value
Age (years), median (IQR^a^)	33.5 (23.5)	24.3 (25.9)	27.1 (27.4)	N/A
**Age group**				**<0.0001^b^**
Younger than 10 years	34 (11.6)	110 (17.1)	144 (15.4)	N/A
10–19 years	22 (7.5)	139 (21.6)	161 (17.2)	N/A
20–29 years	52 (17.7)	172 (26.7)	224 (23.9)	N/A
30–39 years	85 (28.9)	64 (9.9)	149 (15.9)	N/A
40–49 years	44 (15.0)	40 (6.2)	84 (9.0)	N/A
50–59 years	37 (12.6)	41 (6.4)	78 (8.3)	N/A
60 years or older	20 (6.8)	78 (12.1)	98 (10.5)	N/A
Total	294 (100.0)	644 (100.0)	938 (100.0)	N/A
**Gender**				**0.02^b,c^**
Male	183 (62.2)	350 (54.4)	533 (56.8)	N/A
Female	109 (37.1)	289 (44.9)	398 (42.4)	N/A
Transgender/unknown	2 (0.7)	5 (0.8)	7 (0.8)	N/A
Total	294 (100.0)	644 (100.0)	938 (100.0)	N/A

Abbreviations: IQR, interquartile range; N/A, not applicable

^a^ Interquartile range from the 25^th^ to 75^th^ percentile

^b^ Significant at *p*<0.05

^c^ Chi-square value determined using “male” and “female” responses only

Sporadic cases ranged in age from one to 96 years, with a median of 24.3 years (Table 1). Just over half (54.4%, n=350) of sporadic cases were male. Sporadic cases were significantly more likely than outbreak-associated cases to report travel outside of Ontario 15–50 days prior to symptom onset (*p*<0.0001) (**Table 2**).

**Table 2:** Individual risk factors reported by sporadic and outbreak-associated cases of hepatitis A in Ontario, Canada, January 1, 2015, and November 22, 2022

Risk factor	Outbreak-associated cases (%)^a^	Sporadic cases (%)^a^	*p*-value
Anal-oral contact	60 (26.8)	22 (5.4)	<0.0001^b^
Close contact with case	72 (37.1)	61 (14.7)	<0.0001^b^
Diapering a child, or assisting a child or adult with bathroom use	32 (16.1)	37 (8.9)	0.008^b^
Illicit drug use^c^	123 (48.6)	16 (3.6)	<0.0001^b^
Travel outside of Ontario during the incubation period	33 (13.9)	413 (70.4)	<0.0001^b^

^a^ Of those with a “yes” or “no” response available for each risk factor

^b^ Significant at *p*<0.05

^c^ Illicit drug use includes but is not limited to injection drug use

During the study period, five multijurisdictional outbreaks (four national outbreaks, one Ontario-only outbreak) and 13 local (single PHU) outbreaks were reported. These ranged in size from three to 166 cases with a median of four cases. Outbreak-associated cases ranged in age from younger than one year to 80 years, with a median of 33.5 years. Most outbreak-associated cases (28.9%) were aged 30–39 years and most (62.2%) were male (Table 1). One of the five multijurisdictional outbreaks was linked to consumption of a nationally distributed, contaminated frozen fruit product (2016, n=19 Ontario cases) and one large Ontario-only outbreak (2017–2019, n=166 cases linked through HAV genotyping and genetic sequencing; 92% of cases occurring in four PHUs) was linked through case interviewing and outbreak investigation by local PHUs to illicit drug use (reported by 67% of cases), MSM (15%) and person-to-person transmission among individuals experiencing homelessness (27%), in various settings utilized by the under-housed population, including shelters and drop-in centres. The remaining three outbreaks did not have an identified source; however, one was suspected to be associated with a contaminated food product.

Of the 13 locally occurring outbreaks, four (30.8%) were reported to be associated with elementary school settings, three (23.1%) were linked to a food handler or food premises, with the remaining six (46.2%) either having an unspecified source or linked to various settings, including private homes and local group home or shelter settings. Outbreak-associated cases, particularly those older than 18 years, were significantly more likely than sporadic cases to report anal-oral contact, close contact with a case of hepatitis A, illicit drug use and diapering a child or assisting an individual with bathroom use (Table 2). Female outbreak-associated cases were significantly more likely to report diapering a child or assisting with bathroom use compared to males (*p*=0.04). Comparatively, males were significantly more likely to report illicit drug use compared to females (*p*=0.04).

Of 459 cases with a “yes” or “no” response available, 47 (10.2%) reported asymptomatic hepatitis A infection. Of those individuals who reported being asymptomatic, 21.3% were children younger than 10 years of age and 21.3% were aged 60 years or older, with the remainder aged 10–59 years. Of 775 cases with a “yes” or “no” response available, most cases (86.6%, n=671) reported jaundice as a symptom. Of 701 cases with a “yes” or “no” response available, most cases (75.6%; n=530) reported being unimmunized for hepatitis A at the time of case interview.

### Geographic distribution

**Figure 2:**
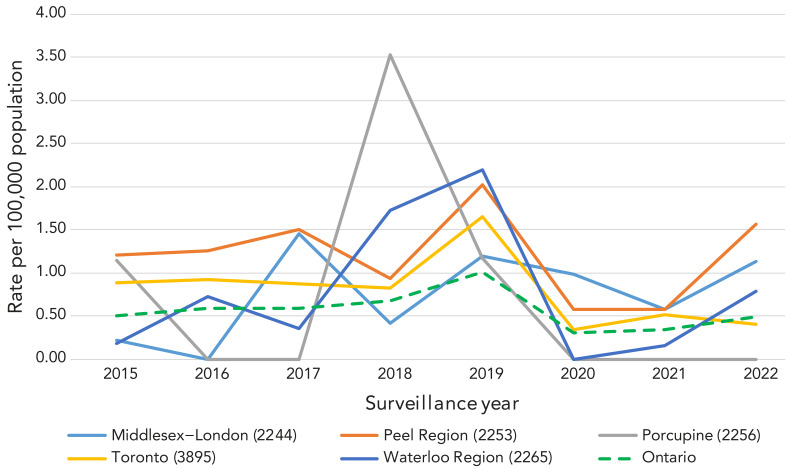
Annual rates of sporadic hepatitis A per 100,000 population, by public health unit and compared to the provincial average, January 1, 2015, to November 22, 2022

Rates of sporadic infection varied by PHU, with the highest rates observed in predominantly urban areas (**Table 3**). Peel Public Health and Toronto Public Health had high rates of infection, above the provincial average, across all study years, with the Region of Waterloo Public Health and Paramedic Services and Middlesex-London Health Unit also having high rates in some years (**Figure 2**). While rates of reported infection in Porcupine Health Unit, located in northern Ontario, were below the provincial average for most years examined, this PHU had the highest rate of sporadic hepatitis A infections in 2018 (Figure 2).

**Table 3:** Annual rates of sporadic hepatitis A infection per 100,000 population, by public health unit and compared to the provincial average, January 1, 2015, to November 22, 2022

Public health unit	Annual rates of sporadic hepatitis A infection (per 100,000 population)								
	2015	2016	2017	2018	2019	2020	2021	2022	Public health unit average
Algoma Public Health	0.0	1.7	0.0	1.7	0.0	0.0	0.0	0.0	0.4
Brant County Health Unit	0.0	0.0	0.0	0.0	1.3	1.3	0.0	0.0	0.3
Chatham-Kent Public Health	0.0	1.0	0.0	0.0	0.0	0.0	0.0	0.0	0.1
City of Hamilton Public Health Services	0.2	0.0	0.4	0.4	0.7	0.2	0.2	0.3	0.3
Durham Region Health Department	0.2	0.8	0.9	0.7	0.9	0.3	0.1	0.0	0.5
Eastern Ontario Health Unit	0.5	0.0	0.0	0.0	0.9	0.0	0.9	0.5	0.4
Grey Bruce Health Unit	0.0	0.6	1.8	0.6	0.6	0.0	0.0	0.0	0.5
Haldimand-Norfolk Health Unit	0.0	1.8	0.0	0.0	0.0	0.0	0.0	0.0	0.2
Haliburton, Kawartha, Pine Ridge District Health Unit	0.0	0.0	0.0	0.5	0.0	0.0	0.5	0.0	0.1
Halton Region Public Health	0.2	0.5	0.2	0.7	1.0	0.5	0.3	0.6	0.5
Hastings Prince Edward Public Health	0.0	0.0	0.6	0.0	0.0	0.0	0.0	0.0	0.1
Huron Perth Public Health	0.0	0.7	0.0	0.0	0.0	0.0	0.7	0.0	0.2
Kingston, Frontenac and Lennox & Addington Public Health	0.0	0.0	0.0	0.0	0.0	0.0	0.5	0.5	0.1
Lambton Public Health	0.0	0.0	0.0	0.0	0.0	0.0	0.0	0.0	0.0
Leeds, Grenville & Lanark District Health Unit	0.0	0.0	0.6	0.0	0.0	0.0	0.0	0.5	0.1
Middlesex-London Health Unit	0.2	0.0	1.5	0.4	1.2	1.0	0.6	1.1	0.8
Niagara Region Public Health	0.4	0.2	0.0	0.2	0.4	0.2	0.2	0.4	0.3
North Bay Parry Sound District Health Unit	0.0	0.0	0.0	0.0	1.5	0.0	0.0	0.0	0.2
Northwestern Health Unit	0.0	0.0	0.0	0.0	0.0	0.0	0.0	0.0	0.0
Ottawa Public Health	0.5	0.3	0.4	0.5	1.0	0.2	0.8	0.5	0.5
Peel Public Health	1.2	1.3	1.5	0.9	2.0	0.6	0.6	1.6	1.2
Peterborough Public Health	0.0	0.0	0.0	2.0	0.0	0.7	0.0	0.0	0.3
Porcupine Health Unit	1.2	0.0	0.0	3.5	1.2	0.0	0.0	0.0	0.7
Public Health Sudbury & Districts	1.0	0.0	0.0	0.5	0.0	0.0	0.0	0.5	0.3
Region of Waterloo Public Health and Emergency Services	0.2	0.7	0.4	1.7	2.2	0.0	0.2	0.8	0.8
Renfrew County and District Health Unit	0.9	0.0	0.0	1.9	0.0	0.0	0.0	0.0	0.4
Simcoe Muskoka District Health Unit	0.0	0.2	0.0	0.5	0.5	0.0	0.2	0.2	0.2
Southwestern Public Health	0.0	1.0	0.0	0.5	0.9	0.0	0.0	0.4	0.4
Thunder Bay District Health Unit	0.6	0.0	0.0	0.0	0.0	0.0	1.3	0.6	0.3
Timiskaming Health Unit	0.0	0.0	0.0	0.0	0.0	0.0	0.0	0.0	0.0
Toronto Public Health	0.9	0.9	0.9	0.8	1.7	0.3	0.5	0.4	0.8
Wellington-Dufferin-Guelph Public Health	0.7	0.7	0.0	1.0	0.3	0.3	0.0	0.3	0.4
Windsor-Essex County Health Unit	0.2	0.7	0.0	0.5	0.2	1.2	0.2	0.9	0.5
York Region Public Health	0.5	0.5	0.7	0.6	0.3	0.2	0.1	0.1	0.4
**Provincial (Ontario) average**	**0.5**	**0.6**	**0.6**	**0.7**	**1.0**	**0.3**	**0.3**	**0.5**	**0.6**

## Discussion

Since 2015, reported cases of hepatitis A in Ontario have increased each year, with the exception of 2020–2022 when case reporting for all diseases of public health significance was impacted by the COVID-19 pandemic (17). Interestingly, almost one third of local outbreaks were associated with an elementary school setting and female outbreak-associated cases were significantly more likely to report diapering a child or assisting an individual with bathroom use. Predominance of females employed in childcare or elementary school settings may have contributed to the observed association between gender and diapering or toileting as a risk factor; however, data regarding case occupation was not available. Children may be more likely to transmit hepatitis A in communal settings such as schools and daycares due to poor hand hygiene and the tendency of infants and younger children to mouth objects (18). Children, including those attending childcare settings, have been linked to the spread of hepatitis A, with attendees and their contacts/relatives at increased risk of infection (4,15,19). The United States Advisory Committee on Immunization Practices recommends HAV vaccination for children aged 12–23 months and unvaccinated children and youth aged two to 18 years (20). Routine vaccination of children in the United States has resulted in HAV infections being rare (20). Currently, Québec is the only jurisdiction in Canada that offers hepatitis A (combined with hepatitis B) vaccine at 18 months of age as part of their routine childhood immunization program (21).

Most cases in our study (76%) were unimmunized for hepatitis A at the time of case interview. Hepatitis A vaccination is not part of the routine immunization schedule in Ontario, however, it is recommended by the National Advisory Committee on Immunization for individuals aged six months and older who are at high risk of exposure or severe outcomes, including travellers to hepatitis A endemic countries, MSM, people who use intravenous drugs and individuals with chronic liver disease (22). Similar to the current literature (5–7,20,23,24), our study found that illicit drug use, close contact with a confirmed case and anal-oral contact were commonly reported risk factors among outbreak-associated cases, potentially indicating suboptimal vaccine uptake by eligible individuals. The finding that male outbreak-associated cases were more likely to report illicit drug use than female outbreak-associated cases was likely influenced by the large Ontario-only outbreak that occurred during the study period (2017–2019), for which illicit drug use was a predominant risk factor and many of the cases (almost two thirds) were male. The United States Advisory Committee on Immunization Practices also recommends routine HAV vaccination for persons experiencing homelessness (20), another important risk factor related to outbreak-associated cases in Ontario.

Sporadic cases were significantly more likely to report travel outside Ontario during the exposure period, with infection in many cases likely acquired during travel. From 2020 to 2022, there was a significant decrease in the number of hepatitis A cases reported in Ontario and elsewhere, with restrictions on travel likely having a substantial impact on travel-associated hepatitis A acquisition rates ((25)). The World Health Organization considers several regions to be endemic for hepatitis A, including most countries in South Asia, South America, Africa, the Middle East and Oceania, with unvaccinated travellers to these areas at increased risk of exposure (22,26). Outbreaks of hepatitis A have previously occurred in Canada and elsewhere following travel to endemic regions (27,28), or due to consumption of contaminated food (10,29); for example, in 2016, 25 cases of hepatitis A linked to consumption of a frozen fruit product were identified from three provinces (30).

Compared to sporadic cases, only a small proportion of outbreak-associated cases reported travel outside of Ontario, indicating local acquisition of infection. Previous studies have explored the under-reporting of hepatitis A in non-endemic countries, including in Canada (27), and noted around 15% of cases may be asymptomatic, contributing to missed opportunities for diagnosis and case reporting, particularly as up to 70%–90% of children younger than six years of age may be asymptomatic (4,31,32). Under-reporting may also occur if symptomatic individuals do not seek medical care or testing. Asymptomatic infections can contribute to undetected transmission of infection and may result in outbreaks, particularly in susceptible populations where most individuals are unimmunized for hepatitis A.

The finding that the highest overall rates of sporadic infection disproportionately occurred within Peel Region and the City of Toronto was likely influenced by the proportion of new immigrants that reside in these areas (33). According to the 2016 census, about 76% of new immigrants to Ontario from 2011 to 2016 settled within the Toronto census metropolitan area, one of the most culturally diverse areas in Canada (33)). Additionally, municipalities within Toronto, Peel Region and the Kitchener-Cambridge-Waterloo areas were among the top 10 census metropolitan areas in Canada with the highest proportion of the population being foreign-born ((33)). Although reported cases of hepatitis A in Ontario are not explicitly asked about recent immigration, those who arrived in Ontario within the 50 days prior to symptom onset would likely have been captured as having travelled during the incubation period. Individuals whose parents or grandparents previously immigrated to Canada from a hepatitis A-endemic country may also be more likely to return to these countries to visit friends and family, increasing their risk of hepatitis A acquisition, particularly if they are not vaccinated for hepatitis A (31)). Prince Edward Island is currently the only Canadian province that identifies immigrants from endemic areas as eligible for publicly funded HAV vaccine (34).

The unusually high rate of sporadic hepatitis A infections in Porcupine Health Unit in 2018 was driven by a small number of cases (fewer than five) in this PHU, which has a small population size compared to other PHUs in Ontario. The high rate of sporadic hepatitis A infections in Middlesex-London Health Unit over the study period was unexpected and was above the provincial average for most years examined. Further investigation may be warranted to ascertain local or other factors that may contribute to observed rates in this region.

### Limitations

This study had several limitations. The data only represented cases reported in iPHIS. As a result, all counts could be subject to varying degrees of under-reporting due to several factors, such as the presence and severity of symptoms, access to health care and healthcare seeking behaviours. Similarly, data for certain risk factors and symptoms may have been incomplete or missing for some cases due to individual case investigator and PHU interviewing and data entry practices. The proportion of cases who were under-housed or homeless or who recently immigrated to Canada are likely underestimated in the dataset as these risk factors are not routinely asked of cases or reported by PHUs. Due to the impact of the COVID-19 pandemic on testing and reporting of diseases of public health significance in Ontario, cases may have been under-ascertained and data for 2020 and 2021 should be interpreted with caution. The standardized questionnaire for hepatitis A only asks cases to self-report if they were ”unimmunized” for hepatitis A at the time of infection, which may be subject to recall bias. For those who report previous vaccination, no information is obtained regarding the number of doses received, date(s) of administration or the reason for vaccination (e.g., post-exposure prophylaxis, anticipated travel). Hepatitis A vaccination data is not routinely entered into a provincial immunization registry; thus, vaccination status could not be verified. Lastly, as this study was intended to be descriptive in nature, analyses were not adjusted to control for potential confounding or effect modification.

## Conclusion

Asymptomatic infection among children and youth in Ontario may be an important contributor to local transmission of HAV within settings such as schools, daycares and private households. While travel to endemic areas for hepatitis A infection increases the risk of sporadic illness, various risk factors, including being under-housed or homeless, using drugs and self-identifying as MSM, may also increase the risk of both acquisition and transmission of infection. Interventions that increase awareness of risk factors and vaccine uptake among individuals at high risk of exposure, including consideration for publicly funded vaccine programs for additional populations (e.g., under-housed persons) and universal vaccination of children, could help to reduce the incidence of hepatitis A infections in Ontario.
